# The impact of social participation on mental health among the older adult in China: an analysis based on the mental frailty index

**DOI:** 10.3389/fpubh.2025.1557513

**Published:** 2025-04-24

**Authors:** Maiyu Jing, Qianqian Wang, Yuheng Jia, Xiaoyong Yu, Kan Tian

**Affiliations:** ^1^School of Health Economics and Management, Nanjing University of Chinese Medicine, Nanjing, China; ^2^School of Elderly Care Services and Management, Nanjing University of Chinese Medicine, Nanjing, China

**Keywords:** social participation, frailty index, mental health, older adults, health

## Abstract

**Objectives:**

Social participation and the psychological well-being of the older adult are correlated. A substantial body of research has delineated the relationship between social participation and the mental health of older adults, while the indicators for assessing the mental health of the older adult are not sufficiently systematic or comprehensive. This study aims to further explore the relationship between social participation and the mental health of older adults from the perspective of mental frailty, providing a reference for the improvement of health among the older adult population.

**Methods:**

We selected 9,208 older adults aged 60 years and older from the China Health and Retirement Longitudinal Study (CHARLS) 2013–2018 database. The mental frailty index was constructed, which integrates both depression and cognitive status to assess the mental health status of the older adult. By using a fixed-effects model, we analyzed the impact of social participation on the mental health of the older adult and compared gender, residence and marriage difference. Meanwhile, the instrumental variables method was used to conduct endogeneity tests.

**Results:**

Overall, 65.8% of the respondents had low social participation. Social participation had a significant enhancement effect on mental health in the older adult (*β* = −0.013, *p* < 0.001). In specific types of social participation, cultural and sports activities (*β* = −0.006, *p* < 0.001), communication and educational activities (*β* = −0.002, *p* < 0.05), and skill-enhancing (*β* = −0.006, *p* < 0.001) activities were negatively correlated with the index of mental frailty of the older adult, whereas volunteering activities (*β* = 0.002, *p* < 0.05) was positively correlated with the index of mental frailty of the older adult.

**Conclusion:**

Social participation has a positive effect on the mental health of the older adult in China, and the effect varies for the older adult with different characteristics, which suggests that social participation may be a protective factor for the mental health of the older adult population. Targeting differentiated population characteristics, individualized policies should be developed for different groups of older persons.

## 1 Introduction

Population aging is a global challenge. As the fastest aging country in the world, China has been affected by aging in terms of social development. According to China’s seventh population census, 18.7% of the population was over 60 years old, and China has already stepped into a deeply aging society (1). As age continues to grow, the inherent self-stereotypes of the older adult deepen, and along with the decline in physical function and cognitive ability. Therefore, the older adult are more prone to negative emotions, and mental health problems come to the fore (2–4). Nearly 30% of older adults in China are at potential risk of depression (2), so it is urgent to improve the mental health of older adults.

Social participation is a broad concept. According to the Activity Theory and Continuity Theory, older people can prolong their previous state of life through social participation and other activities (5), and improve their emotional depression caused by the interruption of their social roles with new participation and new roles, so that they can regain a sense of fulfillment in social participation to shorten the distance between themselves and the community (6–8).

Previous studies have shown a correlation between social participation and mental health in older adults (9, 10). Some studies have shown that regular monthly participation in a certain level of social activities helps to promote health-related quality of life (11). However, due to reduced social contact, loneliness may increase, and with it the risk of cognitive decline may be elevated (12, 13). In addition, among various social activities, participation in recreation activities reduces depression in older adults (14, 15). Older adults can improve their emotional well-being and self-efficacy by participating in various forms of social activities (16, 17). Meanwhile, some studies have drawn other conclusions, some studies found that social participation does not reduce the risk of depression in older adults (18), while others found that social participation may be detrimental to mental health (19, 20). In addition, the assessment of older adults’ mental health in previous studies was faint, with single dimensions such as depression, cognitive ability, or life satisfaction being assessed, and conclusions have not been entirely consistent. Also, the level and type of social participation needs to be further explored. Ageism is prevalent in East Asian cultures, and there is a great deal of variation in the ability of older adults to participate in society as they age, with different characteristics of older adults experiencing different types, intensities, and emotions of social participation (21, 22).Therefore, this study reduced the limitations of traditional research by constructing a mental frailty index that integrates both depression and cognitive ability to systematically and comprehensively measure the mental health of older adults. At the same time, the degree of social participation of the older adult was calculated from the rate and frequency of participation in social activities in a multidimensional manner, and the types of social participation were subdivided to elucidate the relationship between social participation and mental health of the older adult, and to further explore the unique effects of different types of social participation on older adults’ mental health and whether the correlation between the two differed in factors such as gender, marital status, and place of residence.

## 2 Methods

### 2.1 Data sources and sample selection

**Figure 1:**
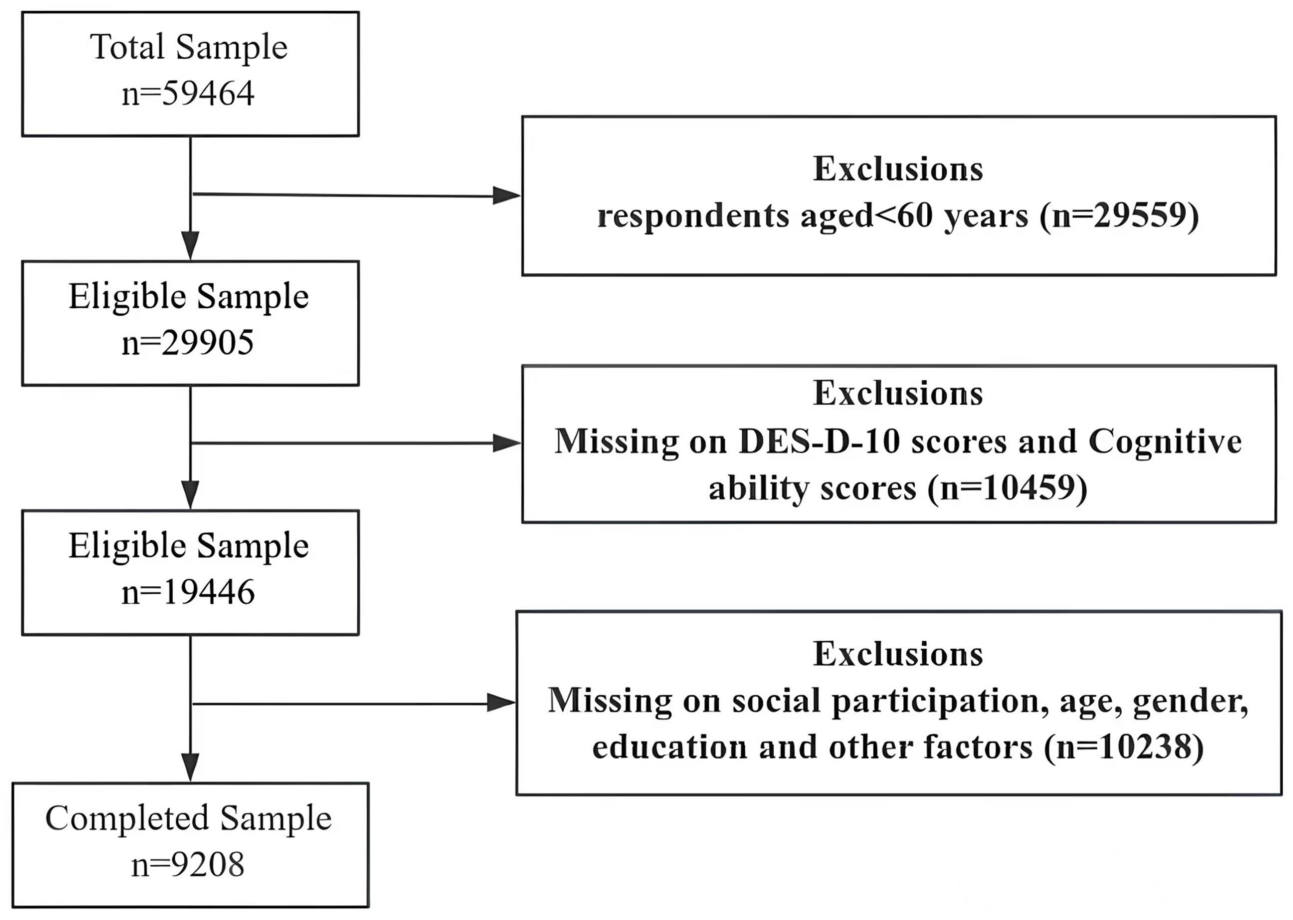
Flowchart of participant selection.

We use the 2013, 2015, and 2018 databases of the China Health and Retirement Longitudinal Study (CHARLS), a large-scale interdisciplinary survey program jointly implemented by Wuhan University and Peking University. The survey includes 28 provinces and autonomous regions across China, and after the national baseline survey in 2011, the national tracking survey will continue in 2013, 2015, 2018, 2020, and 2021–2023. The survey data are representative of the health, family situation, social background, and economic conditions of the older adult, and are used to analyze population aging in China and promote interdisciplinary research on aging. According to the purpose of the study, we chose older adult people aged 60 and above as participants, and a total of 9,208 participants were included. The following sample data were omitted: (i) age < 60 years old; (ii) missing data on CES-D-10 and cognitive ability measures; (iii) missing data on factors such as social participation, age, gender, and education. The participant selection process is shown in Figure 1.

### 2.2 Variables

#### 2.2.1 Dependent variable

The main dependent variable is mental health, and this study reflects the mental health of the older adult by constructing a mental frailty index. The Mental Frailty Index, which was constructed with reference to previous studies and combined with the content of the CHARLS questionnaire, can reflect the mental health status of the older adult in a more comprehensive way (23). The Mental Frailty Index was calculated using the Depression Level and Cognitive Ability scales, both of which take the value of 0–1. Depression level was measured using the Center for Epidemiology Studies Depression Scale (CES-D-10) from the CHARLS questionnaire, which consists of 10 different items that ask older adults to self-assess their psychological feelings and behaviors in the past week. Responses to the 10 items are on a three-point scale: 0 (rarely or not at all), 0.5 (not too much or sometimes), and 1 (most of the time) (24). Cognitive function was assessed using the Modified Mental State Examination (MMSE), which consists of two parts, mental status and situational memory (25). Mental status consisted of a combination of the older adults’ date perception, numeracy, and drawing ability; the date perception and numeracy parts had five questions each, with one point for each question, and the drawing ability, which refers to the ability to correctly draw the pictures seen on a piece of paper, is worth one point, for a total of 11 points in the module of mental status; situational memory was assessed by the number of words respondents could correctly recall after hearing 10 words (26, 27), with one word counting for 1 point, totaling 10 points, and the cognitive ability module totaled 21 points, dividing cognitive ability into five levels: 1 (≤7 points), 0.75 (8–12 points), 0.5 (13–14 points), 0.25 (15–17 points), and 0 (18 points and above).

Frailty index (FI) is the ratio of the number of indicators with health risks to the overall number of indicators in the rating of the constructed health indicators (28). In this study, we constructed the mental frailty index for assessment:


$$\mathrm{Mental frailty index}=\frac{\sum_{j=1}^{N_{j}}D_{j}}{N}$$

Where *N* is the total score of each constructed mental frailty index variable, in this study *N* = 11; D_j_ is the value of the mental frailty index variable corresponding to the jth variable reflecting mental health, D_j_ = 1 means that the jth mental health variable takes the value of unhealthy condition, and D_j_ = 0 means that the jth mental health variable takes the value of healthy condition.

#### 2.2.2 Independent variables

In our study, the social participation was defined as the social activeness of older adults. Social activeness consists of “participation in socialization activities” and “frequency of socialization activities.” Based on the CHARLS questionnaire question “In the past month, did you engage in any of the following socialization activities?,” by assigning each option a value divided into two parts (yes = 1, no = 0) (29), and the frequency of socialization was determined by the responses to the question “How often did you participate in social activities in the past month?,” which were assigned values of “rarely,” “almost every week,” and “almost every day” were assigned values of 1, 2, and 3 (30), respectively, and the social activity level of the older adult was the product of the values assigned to the above two questions (31). Accordingly, the theoretical values of social activeness of the older adult are 0 ~ 33, and the actual values of social activeness of the older adult are 0 ~ 20 based on the responses of the included samples. In our study, social activeness between 0 ~ 2 is defined as a low level of social activeness, and social activeness between 3 ~ 20 is defined as a high level of social activeness. At the same time, according to the nature of social activities, they were categorized into four types: cultural and sports activities, communication and educational activities, skill-enhancing activities, and volunteering activities. The cultural and sports activities included “dancing, working out, practicing qigong, etc.,” the communication and educational activities included “visiting friends, socializing with friends; playing mahjong, chess, cards, and going to the Community Room,” and the skill-enhancing activities embodied “attending school or training courses; speculating on stocks; and surfing the Internet,” and volunteering activities included “participating in volunteer or charitable activities; providing help to family members, friends or neighbors with whom you do not live; taking care of patients or people with disabilities with whom you do not live; participating in activities of associations.” The activity level of each of the four different social types was calculated according to the social activity calculation method.

#### 2.2.3 Control variables

We chose three types of control variables including demographic characteristics, socioeconomic support, and health behavioral factors; demographic characteristics included gender (female, male), age (60–69, 70–79, ≥80), education (primary and below, elementary, middle school, high school and above), marital status (unmarried, married), and residence (rural, urban); socioeconomic support included pension insurance (no, yes), health insurance (no, yes); health behaviors included smoking (no, yes), alcohol use (no, yes), exercise (no, yes), sleep duration (<6, 6–6.9, 7–7.9, ≥8), and number of chronic diseases. We defined “married and living with spouse” and “married but not living with spouse for the time being” as married; correspondingly, “separated” “divorced” “widowed” and “never married” constituted unmarried.

### 2.3 Statistical analysis

Data were cleaned and statistically analyzed using Stata 16.0, with the mean and standard deviation used for the continuous variables, frequency and percentage for the categorical variables. The Chi-square test was used for between-group comparisons of categorical variables, and t-tests was for continuous variables. To determine the relationship between social participation and older adults’ mental health, we used Fixed-Effects Model (FEM) for data analysis. Fixed effects regression is a class of variables that vary with individuals but not over time in a spatial panel data approach that eliminates endogeneity problems from omitted variables by controlling for individual characteristics correlated with the dependent variable but unobserved (32–34). It is worth noting that social participation may be influenced by individual, family, and environmental factors, so we further addressed sample endogeneity using an instrumental variable approach. Finally, we tested the robustness of the results by replacing the independent variables and adjusting the control variables, and further explored whether there are differences in the effects of different types of social participation on the mental health of different older adult groups by analyzing the heterogeneity among gender, marital status, and residence. Statistical differences were considered significant when *p* < 0.05.

## 3 Results

### 3.1 Descriptive analysis of the basic characteristics of the older adult

Table 1 describes the basic characteristics of the older adult. A total of 9,208 older adults aged 60 years and above were included as participants. Demographically, it is dominated by 60-79-year-olds (95.39%) and married seniors (84.88%), with a slight majority of males (56.85%), and most of the sample had a primary school level of education or less (65.81%). In terms of social security, most of the older adult were covered by medical insurance (97.45%) and pension insurance (89.42%). With regard to health, the older adult had relatively healthy living habits, but basically suffered from more than two chronic diseases on average (2.34 ± 1.88), and the mean value of the mental frailty index was 0.32. In terms of social participation, the social activeness (65.78%) of the older adult was generally low, and the type of socialization with the highest social activeness (1.15 ± 1.56) was the communication and educational activity. For the outcome variables, social activeness (*p* < 0.001), type of social participation (*p* < 0.001), gender (*p* < 0.001), education (*p* < 0.001), marital status (*p* < 0.001), residence (*p* < 0.001), smoking (*p* = 0.002), sleep duration (*p* < 0.001), and number of chronic diseases (*p* < 0.001) were statistically significantly different.

**Table 1:** Basic characteristics of old adults (*n* = 9,208).

(A)		
Variables	Total number	*p*-values
	*N* (%)	
Social activeness		<0.001
Low-level	6,057 (65.78)	
High-level	3,151 (34.22)	
Types of social activity		<0.001
Cultural and sports activities	0.201 (0.73)	
Communication and educational activities	1.15 (1.56)	
Skill-enhancing activities	0.22 (0.81)	
Volunteering activities	0.28 (0.79)	
Age(year)		<0.001
60–69	6,207 (67.41)	
70–79	2,576 (27.98)	
>80	425 (4.62)	
Gender		<0.001
Woman	3,973 (43.15)	
Man	5,235 (56.85)	
Education		<0.001
Primary and below	3,470 (37.68)	
Elementary	2,590 (28.13)	
Middle school	1964 (21.33)	
High school and above	1,184 (12.86)	
Marital status		<0.001
Married	1,392 (15.12)	
Unmarried	7,816 (84.88)	
Residence		<0.001
Urban	3,913 (42.50)	
Rural	5,295 (57.50)	
Pension insurance		0.161
No	974 (10.58)	
Yes	8,234 (89.42)	
Health insurance		0.337
No	235 (2.55)	
Yes	8,973 (97.45)	
Smoking		0.002
No	6,485 (70.43)	
Yes	2,723 (29.57)	
Alcohol use		0.224
No	5,816 (63.16)	
Yes	3,392 (36.84)	
Exercise		0.596
No	803 (8.72)	
Yes	8,405 (91.28)	
Sleep duration(h)^a^		<0.001
<6	3,192 (34.67)	
6–6.9	2093 (22.73)	
7–7.9	1,571 (17.06)	
≥8	2,352 (25.54)	

(B)		
Variables	Mean (SD)	*p*-values
Number of chronic diseases	2.34 (1.88)	<0.001
Mental frailty index	0.32 (0.16)	

N.B. Total percentages of some variables are not equal to 100 due to rounding.

^a^Actual hours of sleep participants got at night (average hours for one night) during the past month. SD, standard deviation.

**Figure 2:**
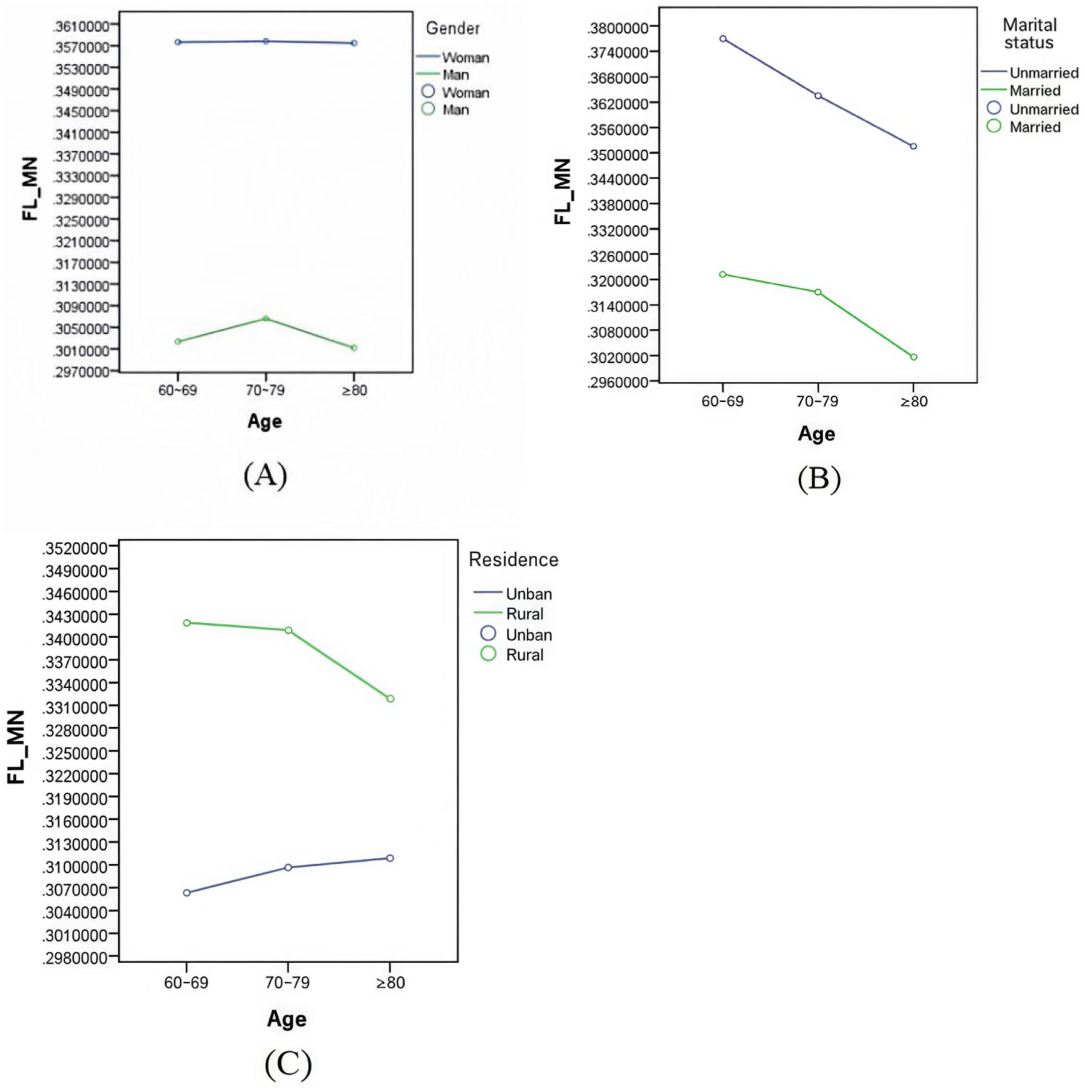
Changes in mental frailty indexes among older adults in different age groups by gender **(A)**, marital status **(B)**, and residence **(C)**.

Figure 2 shows the changes in mental frailty index for various age groups of older adults by gender (A), marital status (B) and residence (C), respectively. It can be seen from the figure that the mean values of mental frailty index were higher for all three age groups: female, unmarried and rural. Married, unmarried and rural older adult showed a decreasing trend in mental frailty index with age, whereas the peak values of mental frailty index for the male older adult group were in the range of 70–79 years.

### 3.2 The relationship between social participation and the mental health of older adults

The impact of social participation on mental health of the older adult based on Fixed-Effects Model regression are shown in Table 2. Model 1 was regressed with the including only the independent variable of social activeness, Model 2, Model 3, and Model 4 sequentially added control variables for basic demographic characteristics, socioeconomic support, and health behaviors of older adults. The regression results showed that the mental frailty index of the older adult was significantly and negatively correlated with social activeness (*β* = −0.013, *p* < 0.01). Among them, gender, marital status, residence, education, and pension insurance all had a significant negative effect on mental frailty among the older adult, compared with smoking, sleep duration, and the number of chronic diseases, which all had a significant positive effect on mental frailty among the older adult. Meanwhile, after adding more control variable, the correlation coefficients gradually leveled off and the regression coefficients became smaller.

**Table 2:** Regression results for social participation and mental health in older adults (*n* = 9,208).

Variables	Model 1	Model 2	Model 3	Model 4
	β (95% CI)	β (95% CI)	β (95% CI)	β (95% CI)
Social activeness	−0.019^***^ (−0.025,-0.012)	−0.023^***^ (−0.020, −0.006)	−0.017^***^ (−0.020, −0.006)	−0.013^***^ (−0.019, −0.006)
Gender		−0.046^***^ (−0.053, −0.039)	−0.047^***^ (−0.059, −0.042)	−0.043^***^ (−0.051, −0.035)
Marital status		−0.038^***^ (−0.047, −0.029)	−0.038^***^ (−0.047, −0.028)	−0.034^***^ (−0.043, −0.025)
Residence		0.027^***^ (0.020, 0.035)	0.027^***^ (0.020, 0.034)	0.031^***^ (0.024, 0.038)
Education		−0.016^***^ (−0.019, −0.013)	−0.016^***^ (−0.020, −0.012)	−0.016^***^ (−0.020, −0.013)
Age(year)		−0.004 (−0.010, 0.002)	−0.004 (−0.010, 0.002)	−0.007^**^ (−0.012, 0.002)
Health insurance			−0.009 (−0.028, 0.010)	−0.017^*^ (−0.036, 0.001)
Pension insurance			−0.010^**^ (−0.002, −0.008)	−0.013^***^ (−0.023, −0.004)
Alcohol use				0.001 (−0.006, 0.008)
Smoking				0.017^***^ (0.009, 0.024)
Sleep duration(h)^a^				−0.018^***^ (−0.021, 0.016)
Exercise				−0.003 (−0.013, 0.008)
Number of chronic diseases				0.017^***^ (0.015, 0.019)

CI, confidence interval. ^a^Actual hours of sleep participants got at night (average hours for one night) during the past month. *p < 0.1, ^**^p < 0.05, ^***^p < 0.01.

### 3.3 The impact of different types of social participation on the mental health of older adults

To explore whether different types of social participation have different effects on the mental health of older people, we regressed the four types of social participation on the mental frailty index. Table 3 presents that the mental frailty index of older adults was significantly negatively associated with the cultural and sports activities, the communication and educational activities, and the skill-enhancing activities, but the volunteering activities had a positive mental frailty influence on index of older adults (*β* = 0.002, *p* < 0.05). This suggests that volunteering not only has no effect on improving the mental health of older adults, but may even exacerbate their mental frailty.

**Table 3:** Regression results for different types of Social participation and mental health in older adults (*n* = 9,208).

Variables	Model 5			
	β (95% CI)	β (95% CI)	β (95% CI)	β(95% CI)
Cultural and sports	−0.006^***^ (−0.010, −0.002)			
Communication and educational		-0.002^**^ (−0.004, −0.001)		
Skill-enhancing			−0.006^***^ (−0.010, −0.002)	
Volunteering				0.002^**^ (0.001, 0.006)
Control variables	Controlled	Controlled	Controlled	Controlled

CI, confidence interval. ^**^p < 0.05, ^***^p < 0.01.

### 3.4 Results of subgroup analysis

Our study conducted subgroup regressions by gender, marital status, residence, pension insurance and exercise to investigate the effects of social participation on the mental health of older adults. Table 4 (A) displays that social participation had a more significant effect on the mental health level of older adult women, compared to men. The positive effect of social participation on mental health was greater for rural participants than for urban ones. Furthermore, social participation had a significant effect on the mental health of unmarried older adult people. For married older adult people, no significant association was found. In Table 4 (B) we can see that there is a difference in the impact of having a pension insurance on the mental health of older adults. Social participation has a significant negative impact on mental frailty in older people who have a pension insurance, compared to those who do not have a pension insurance. In addition, exercising or not exercising had no effect on the association between social participation and mental health in older adults.

**Table 4:** Results of a heterogeneous analysis of the impact of social participation on the mental health of older adults.

(A)						
Variables	(1) Woman	(2) Man	(3) Unmarried	(4) Married	(5) Rural	(6) Urban
	β (95% CI)	β (95% CI)	β (95% CI)	β (95% CI)	β (95% CI)	β (95% CI)
Social activeness	−0.021^***^ (−0.031, −0.011)	−0.006 (−0.018, 0.002)	−0.034^***^ (−0.053, −0.016)	−0.009 (−0.016, −0.002)	−0.017^***^ (−0.026, −0.009)	-0.009^*^ (−0.018, 0.001)
Control variables	Controlled	Controlled	Controlled	Controlled	Controlled	Controlled
Cons.	0.449^***^ (0.405, 0.494)	0.000 (.)	0.459^***^ (0.383, 0.535)	0.000 (.)	0.438^***^ (0.396, 0.480)	0.443^***^ (0.406, 0.481)
Sample number	3,973	5,235	1,392	7,816	3,913	5,295
R^2^ (adjusted)	0.152	0.103	0.141	0.139	0.151	0.136

(B)				
Variables	(7) No pension insurance	(8) Have pension insurance	(9) No exercise	(10) Exercise
	β (95% CI)	β (95% CI)	β (95% CI)	β (95% CI)
Social activeness	−0.015 (−0.037,0.010)	−0.012^***^ (−0.020,-0.006)	−0.035^***^ (−0.060,-0.010)	−0.011^***^ (−0.017,-0.004)
Control variables	controlled	controlled	controlled	controlled
Cons.	0.432^***^ (0.354,0.510)	0.000 (.)	0.360^***^ (0.267,0.453)	0.000 (.)
Sample number	974	8,234	803	8,405
R^2^(adjusted)	0.172	0.154	0.201	0.144

Cons, constant term (math.); CI, confidence interval. ^***^p < 0.01, *p < 0.1.

R^2^(adjusted): R^2^ is used to measure the extent to which the independent variables explain the variation in the dependent variable, and the adjusted R^2^ more accurately reflects the goodness of fit of the model.

Table 5 (A) shows that the effects of different types of social participation on the mental health of older adults of different gender. There was no difference in the effects of skill-enhancing activities on the mental health of older people of different gender, and that cultural and sports activities and communication and educational activities had more significant negative effects on mental frailty of female older people, while volunteering activities had significant positive effects on mental frailty of male older people. Among older adults of different marital status, only communication and educational activities and volunteering activities had differential effects on their mental health, with communication and educational activities having a significant negative effect on mental frailty of unmarried older adults. As well volunteering activities had a significant positive effect on mental frailty of married older adults. Inconsistent with the results of the heterogeneity analysis of the effect of total social activities on mental frailty of older adults, cultural and sports activities, communication and educational activities, and skill-enhancing activities had a significant negative effect on mental frailty of older adults in rural areas, while there was no significant difference between urban and rural older adults in the effect of volunteering activities on their mental health. In Table 5 (B) we can see that regarding the availability of pension insurance, only communication and educational activities had differential effects on the mental health of older adults. In terms of whether or not they exercised, the results were reversed. With the exception of communication and educational activities, the other three types of social activities had a heterogeneous effect on the mental health of older adults.

**Table 5:** Results of a heterogeneous analysis of the impact of different types of social participation on the mental health of older adults.

(A)						
Variables	(1) Woman	(2) Man	(3) Unmarried	(4) Married	(5) Rural	(6) Urban
	β (95% CI)	β (95% CI)	β (95% CI)	β (95% CI)	β (95% CI)	β (95% CI)
Cultural and sports	−0.008^***^ (−0.013, −0.002)	-0.002 (−0.008, 0.005)	−0.012^**^ (−0.022, 0.001)	−0.005^**^ (−0.009, −0.001)	−0.006^***^ (−0.010, −0.001)	−0.005 (−0.013, 0.003)
Communication and educational	−0.003^**^ (−0.006, 0.001)	−0.001 (−0.004, 0.001)	−0.007^**^ (−0.022, 0.001)	−0.001 (−0.003, 0.001)	−0.004^***^ (−0.006, −0.001)	-0.001 (−0.004, 0.002)
Skill-enhancing	−0.009^***^ (−0.015, −0.002)	−0.005^*^ (−0.010, −0.001)	−0.011^*^ (−0.023, 0.004)	−0.006^***^ (−0.010, −0.002)	−0.006^***^ (−0.010, −0.001)	−0.006 (−0.016, 0.003)
Volunteering	−0.002 (−0.005, 0.007)	0.006^**^ (0.002, 0.012)	−0.007 (−0.023, 0.004)	0.004^*^ (0.001, 0.010)	0.001 (−0.001, 0.008)	0.004 (−0.001, 0.011)
Control variables	Controlled	Controlled	Controlled	Controlled	Controlled	Controlled
Cons.	0.449^***^ (0.405, 0.494)	0.000 (.)	0.459^***^ (0.383, 0.535)	0.000 (.)	0.438^***^ (0.396, 0.480)	0.443^***^ (0.406, 0.481)
Sample number	3,973	5,235	1,392	7,816	3,913	5,295
R^2^ (adjusted)	0.152	0.103	0.141	0.139	0.151	0.136

(B)				
Variables	(7) No pension insurance	(8) Have pension insurance	(9) No exercise	(10) Exercise
	β (95% CI)	β (95% CI)	β (95% CI)	β (95% CI)
Cultural and sports	−0.012^*^ (−0.026, 0.002)	−0.006^***^ (−0.011, −0.002)	−0.007 (−0.038, 0.024)	−0.007^***^ (−0.011, −0.003)
Communication and educational	−0.002 (−0.009, 0.004)	−0.003^**^ (−0.004, −0.001)	−0.008^**^ (−0.015, −0.001)	−0.002^*^ (−0.004, 0.001)
Skill-enhancing	−0.014^*^ (−0.030, 0.002)	−0.009^***^ (−0.013, −0.005)	−0.009 (−0.028, 0.011)	−0.009^***^ (−0.013,0.005)
Volunteering	0.008 (−0.005, 0.020)	0.004 (−0.001, 0.007)	0.003 (−0.024, 0.019)	0.004^*^ (0.001, 0.008)
Control variables	Controlled	Controlled	Controlled	Controlled
Cons.	0.384^***^ (0.318, 0.450)	0.358^***^ (0.331, 0.384)	0.326^***^ (0.247, 0.405)	0.377^***^ (0.352, 0.402)
Sample number	974	8,234	803	8,405
R^2^ (adjusted)	0.167	0.138	0.196	0.136

Cons, constant term (math). ^*^p < 0.1, ^**^p < 0.05, ^***^p < 0.01.

R^2^(adjusted): R^2^ is used to measure the extent to which the independent variables explain the variation in the dependent variable, and the adjusted R^2^ more accurately reflects the goodness of fit of the model.

### 3.5 Robustness check

To further ensure the robustness of the regression results, we tested the robustness of the regression results of each model by both replacing the independent variables and adjusting the control variables.

Firstly, we replaced the independent variables and used “whether to participate in social activities” as the proxy variable for “social participation,” which was estimated using the fixed-effect model, among them, Model 6–9 displayed the regression results after replacing the independent variables and adding demographic characteristics, social and economic support, health behavior variables in turn. As shown in Table 6, whether or not to participate in social activities has a significant negative effect on mental frailty of the older adult. In addition, we replaced mental frailty index with life satisfaction and added the original control variables to the analysis. Model 10 showed that social participation has a significant positive effect on life satisfaction, and this result was consistent with the original findings.

**Table 6:** Results of robustness check performed by replacing the independent variables, dependent variables (*n* = 9,208).

Variables	Model 6	Model 7	Model 8	Model 9	Model 10^a^
	β (95% CI)	β (95% CI)	β (95% CI)	β(95% CI)	β (95% CI)
Whether to participate in social activities	−0.012^***^ (−0.015, −0.008)	−0.015^***^ (−0.019, −0.008)	−0.009^***^ (−0.011, −0.006)	−0.007^***^ (−0.010, −0.004)	
Social activeness					0.057^***^ (0.024, 0.089)
Basic demographic characteristics	Uncontrolled	Controlled	Controlled	Controlled	Controlled
Socioeconomic support	Uncontrolled	Uncontrolled	Controlled	Controlled	Controlled
Health behaviors	Uncontrolled	Uncontrolled	Uncontrolled	Controlled	Controlled
Cons.	0.296^***^ (0.288, 0.304)	0.370^***^ (0.354, 0.386)	0.368^***^ (0.352, 0.385)	0.407^***^ (0.380, 0.435)	2.766^***^ (2.619, 2.913)
R^2^(adjusted)	0.014	0.007	0.008	0.015	0.035

Cons, constant term (math).

^a^Model 10 is the result of a fixed-effects model conducted by replacing mental frailty index with life satisfaction. ^***^p < 0.01.

R^2^(adjusted): R^2^ is used to measure the extent to which the independent variables explain the variation in the dependent variable, and the adjusted R^2^ more accurately reflects the goodness of fit of the model.

Secondly, we adjusted the control variables by adding “Number of living children” and “Disabled or not” to the regression model of the effect of social activeness on mental frailty of the older adult, and we found that social activeness still has a significant negative effect on mental frailty of the older adult, as shown in Table 7. Between the two different tests, the relative relationship between the coefficients of social participation and the mental frailty index of the older adult and the significance level of the coefficients did not change significantly. The consistency suggested strong support for the reliability of the regression results.

**Table 7:** Results of a fixed effects model of the impact of social participation on the mental health of older adults after adding control variables.

Variables	Model 10	Model 11
	β (95% CI)	β (95% CI)
Social activeness	−0.012^***^ (−0.019, −0.006)	−0.010^***^ (−0.017, −0.004)
Number of living children	0.005^***^ (0.002, 0.007)	
Disabled or not		0.039^***^ (0.032, 0.045)
Control variables	Controlled	Controlled
Cons.	0.411^***^ (0.382, 0.440)	0.407^***^ (0.378, 0.435)
Sample number	9,208	9,038
R^2^(adjusted)	0.149	0.162

Cons, constant term (math). ^***^p < 0.01.

R^2^(adjusted): R^2^ is used to measure the extent to which the independent variables explain the variation in the dependent variable, and the adjusted R^2^ more accurately reflects the goodness of fit of the model.

### 3.6 Endogeneity test

The aim of this study was to investigate the impact of social participation on the mental health of older people. However, older people’s psychological condition also affects their social participation, so there may be a bidirectional causal relationship between social participation and older people’s mental health, which in turn creates endogenous problems. To identify the causal effect of social participation on the mental health of older adults, a two-stage ordinary least squares method with instrumental variables is added (Table 8). We selected older adults’ social relationships as an instrumental variable, measured indirectly using the CHARLS questionnaire, “If you needed care in your daily life in the future, would a family member or friend be able to take care of you in the long term?”.

**Table 8:** An endogeneity test of the effect of social participation on the mental health of older adults.

Variables	Stage 1		Stage 2^a^	
	β (95% CI)	SD	β (95% CI)	SD
Cons.	1.293^***^ (1.201, 1.386)	0.046	1.008^***^ (0.753, 1.263)	0.142
Social activeness			−0.485^***^ (−0.693, −0.277)	0.105
Whether to have relatives or friends who can take care of you for a long time (instrumental variable) SW-F statistic	−0.059^***^ (−0.079, −0.037)	−5.395		
	30.34		28.48	

SD, standard deviations; Cons, constant term (math.). Control variables were controlled in stage 2 and adjusted r^2^ = 0.265. ^***^p < 0.01.

On the one hand, the social relationships of the older adult had a certain inducing effect on whether they participate in social activities or not; on the other hand, the social relationships of the older adult were not directly related to their own health, so this variable is a more appropriate instrumental variable. In the two-stage least squares estimation of the instrumental variable, the results of the first stage showed that “Whether to have relatives or friends who can take care of you for a long time” had an effect at the 0.01 level, which is a good explanation for the endogenous explanatory variables. The results of stage 2 presented that social activeness still had a negative effect on mental frailty of older adults after the addition of the instrumental variable, and the regression coefficient of the effect of social activeness on mental frailty of older adults increased from 0.013 to 0.493 after the addition of the instrumental variable, indicating that if the instrumental variable is not used to solve the endogeneity problem, the effect of social participation on the mental health of older adults will be underestimated.

In addition, the instrumental variables passed a series of statistical tests. On the one hand, the Hausman Test showed *p* < 0.001, which proved that there is an endogeneity problem between social participation and mental health of the older adult; On the other hand, the value of F-statistic in stage 1 was 30.34, which was higher than the critical value of 16.38 proposed by Stock-Yog, which indicated that there was no weak instrumental variable and the hypothesis of correlation was satisfied. The above test indicated that the instrumental variables selected for this study are valid.

## 4 Discussion

Using longitudinal data from three consecutive periods of the China Health and Retirement Longitudinal Study (CHARLS) in 2013, 2015, and 2018, we identified the effects of social participation and different types of social participation on the mental health of older adults. We produced three main findings in this study. First, our older adults have poorer mental health and lower levels of social participation (35). Overall, social participation has a positive effect on the mental health of older adults. Second, the effects of different types of social participation on the mental health of older adults were not entirely consistent, with cultural and sports activities, communication and educational activities, and skill-enhancing activities improving the mental health of older adults, while volunteering activities may be detrimental to the mental health of older adults. Third, there are gender, marital status and pension insurance differences in the impact of social participation on the mental health of older adults. The association between social participation and better mental health is more pronounced for unmarried and female older adults with pension insurance than for male and married older adults without pension insurance (36, 37).

This study showed that the social participation of older adults in our country is generally low, with those with low levels of social participation accounting for 65.78% of the total sample. In addition, the mental health status of the older adult differed significantly by gender, with the female older adult population being more in need of improvement, which is consistent with previous studies. This may be due to the fact that women have greater emotional needs, their work-related interpersonal networks gradually shrink after retirement, and changes in their social roles increase their psychological distress (38). At the same time, perhaps due to widowhood and children working elsewhere, older women lack the companionship of their spouses and children (39), and their sense of loneliness deepens. Therefore, more attention should be paid to the mental health status of older women, especially unmarried women who lack the companionship of spouses and children.

Social participation has a significant positive effect on the mental health of the older adult, and this finding is consistent with the existing literature (39, 40). As the older adult face role changes and lifestyle changes after retirement, the sudden changes will make them unable to adapt quickly and suffer from a huge psychological gap (41). Therefore, appropriate social activities can help them adapt to the new pace of life as soon as possible, enrich the content of their lives and increase the life satisfaction. In terms of different types of social participation, cultural and sports activities, communication and educational activities, and skill-enhancing activities could improve the psychological health of the older adult, while volunteering activities were likely to be detrimental to the psychological health of the older adult, especially the married, male older adult group. This contradicts previous research findings. Although most studies have found that participation in volunteer activities improves older adults’ mental health (42, 43), the frequency of participation and group differences need further characterization and attention. Frequent participation in volunteer activities may lead to excessive physical exertion and work-relatedness (44, 45). Increased levels of volunteering are subsequently associated with reductions in life satisfaction (46). Meanwhile, due to the role division in traditional Chinese culture, compared with women, men’s socialization tends to devote more time and energy to their career, a gender difference that prevents men from taking on the same level of caregiving responsibilities in the home (47, 48). So participating in volunteer-based social activities is more challenging for older men who are not as likely to gain emotional support and satisfaction from these volunteer activities, thus causing poorer mental health. Therefore, in improving and optimizing the level of social participation of older persons, attention should be paid to the types of social participation of different groups, changing the stereotypes of older persons themselves and broadening the path of social participation for older adults.

In addition, our study also provides extensive information on heterogeneity. The data from this study showed that social participation had a more significant effect on improving the mental health of the female, unmarried and having pension insurance older age group (37, 49, 50). These findings are similar with findings from previous studies. This difference can be attributed to several key factors. First, differences in the social division of labor and family responsibilities. Women are more likely than men to be responsible for domestic work and family responsibilities, including intergenerational support for their children, which are also more likely to have a negative impact on older women’s mental health. Therefore, on the basis of lower levels of mental health, participation in social activities allows women to have a broader source of emotional support (51), and women are also more likely to make new friends through social activities, rich social activities can promote them to share their lives and have emotional exchanges, which can improve mental health. Second, there are differences in social support networks. Marriage has greater impact on health in old age, and the older adult have become accustomed to the mutual support of marriage in daily life, while divorce, widowhood, separation, will lead to the lack of stable spiritual care of one of the parties in the marriage, such as the care and companionship of the original spouse, and therefore they need more to establish and maintain social ties through social participation to obtain emotional support and satisfaction. Third, pension insurance provides older people with a stable source of income, relieves financial pressure and reduces their financial dependence on their families, making them more able and willing to participate in social activities (37, 52, 53).

This study shows that social participation has a positive effect on the mental health of the older adult population, in which the cultural and sports activities, communication and educational activities, and skill-enhancing activities can improve the mental health of the older adult, while the volunteering activities may be detrimental to the mental health of the older adult. This study provides valuable insights into enriching and differentiating the types of social participation to improve the mental health of older adults. In addition, the results of robustness and endogeneity tests are consistent with the main findings of this study, which can ensure the accuracy and validity of the research construct.

Despite the strengths of this study, there are several limitations. First, this study was based on data from a large longitudinal study, and the self-report questionnaire used did not completely eliminate the possibility of recall bias. Second, due to the longitudinal nature of this study, there were some sample losses due to factors such as missing interviews and missing information, which may have led to selection bias. Finally, the selection of control variables could not cover all the factors that affect the mental health of older people, but only as many of the more critical factors as possible. The scales used to assess depression and cognitive ability in older adults do not represent realistic diagnoses. In the future, we will deepen our study through further mechanistic investigations.

## 5 Conclusion

Our study is one of the few longitudinal studies in China to describe the mental health of older adults by constructing a mental frailty index. In accordance with our findings, there is a significantly positive association between Social participation and mental health in Chinese older adult, and the impact of different types of social participation on older people’s mental health varies across different genders, marriage, residence and exercise. This suggests that policies to increase social participation of old adults are essential. In accordance with China’s unique social and cultural norms, older people should be encouraged to change their own perceptions, keep up with the times with a positive attitude, and rebuild their ties with society. In addition, communities can make use of geriatric social activity platforms to provide diversified opportunities for social participation for different types and levels of older adult people, while further improving community aging renovation and creating environmentally friendly communities, so as to achieve positive aging.

## Data Availability

The original contributions presented in the study are publicly available. This data can be found at: https://charls.charlsdata.com/pages/data/111/zh-cn.html.
